# Twelve tips for teaching twelve transferable skills

**DOI:** 10.15694/mep.2018.0000177.1

**Published:** 2018-08-21

**Authors:** Helen R Watson, Steven Burr

**Affiliations:** 1 Peninsula Medical School

**Keywords:** Generic skill, soft skill, key skill, content knowledge, knowledge recall

## Abstract

This article was migrated. The article was marked as recommended.

There is an increasing awareness of the importance of transferable skills. With the internet now allowing almost instant access to information, the recall of factual knowledge no longer has the primacy it has held for centuries. In contrast, transferable skills remain particularly important for medical students because they receive a generic education in preparation for a diverse range of careers. The twelve tips in this article are more relevant for certain skills than others. Transferable skills should be taught in context and students should be made aware of how the skills will play a part in their future. Learning outcomes must be made clear to both staff and students, and without clear assessment of transferable skills students are likely to focus exclusively on knowledge. Students and staff should proactively engage with transferable skills training, rather than taking a purely remedial approach.

## Introduction

Transferable skills ( Table 1) are demanded by employers to varying extents in all areas of work ( Fallows, 2000).  The aspects of practice where new doctors and their supervisors report being least prepared for practice are often those encompassed by transferable skills (e.g. interpersonal aspects of practice; Monrouxe et al., 2014).  Medical education and training increasingly needs to prioritise developing the generic qualities required to function as a professional, alongside developing the ability to adapt to changing requirements throughout a career.  The difficulty with this is that transferable skills can be intangible; and challenging to establish constructive alignment from teaching to assessment in order to provide both motivation for, and confirmation of, achievement ( Burr and Brodier, 2010).  The ultimate aim is preparation for practice as an ‘ongoing endeavour’, focused on the endpoint of being fully responsible and accountable for providing safe and effective patient care ( Monrouxe et al., 2014).  To achieve this it is necessary to be proactive in responding to the different starting points of students, as well as changes in clinical practice ( Watson and Burr, 2018).  With this in mind, we have identified twelve curricular considerations for optimising the development of transferable skills.

## Tip 1 - Identify the skills required

It is necessary to predict what transferable skills any student might need over the course of their medical career.  It is important to establish a clear purpose and define learning outcomes to frame everything that follows, along with an acknowledgement that requirements may need to change over time. It is also necessary to ensure constructive alignment between developing the skills and their assessment.  First confirm which transferable skills ( Table 1) are needed to be mandatory for practice (e.g. AMC, 2012; CACMS, 2015; GMC, 2018; ACGME, 2017; and KNMG, 2017), and then the extent of mastery required for the stage of training.  For example, we could expect a first year student to be able to locate several review papers on a topic, and draw conclusions based on these. In later years, we may expect a more thorough interrogation of the literature with critical appraisal and novel recommendations.  Once identified, the transferable skills and the level of mastery required should be articulated as Learning Outcomes that are transparent to both students and staff.

**Table 1. Twelve transferable skills relevant to medical training. ** Some are more tangible and easier to teach and/or assess than others, and there is also overlap between some skills (e.g. being professional and an effective team member).  Some skills are more important than others for different specialties (e.g. differing communication requirements for psychiatry and pathology) and at different stages of a career (e.g. leadership requirements with advancing seniority; Dijkstra et al., 2014).

**Table:** 

1.	Professionalism (public service, compassion, ethical and legal acumen)
2.	Reflective practice (self-awareness, self-improvement)
3.	Resilience (coping with stress, dealing with uncertainty, adjusting to transitions/adapting to change)
4.	Communication (feedback and networking)
5.	Cultural consciousness (equality, empathy, emotional intelligence)
6.	Team working (leadership, interprofessionalism, collegiality, collaboration, insight into health and safety of self and colleagues)
7.	Planning, organisation and timekeeping (management, prioritisation, multitasking)
8.	Technical/IT (adopting modern innovations, technical literacy)
9.	Life-long learning (long-term Self-Directed Learning)
10.	Research (searching sources for information, critical evaluation, synthesis, presenting evidence, qualitative and quantitative literacy)
11.	Problem solving and improvisation (analysis and intuition, calculation and numeracy)
12.	Decision making (initiative, cognizance of bias, moral dilemmas, acting on own limitations)

## Tip 2 - Rank the importance of, and prioritise, skills training

The learning outcomes should be given prominence according to need; both relative to other transferable skills and relative to other aspects of the curriculum ( Whittle and Murdoch-Eaton, 2001).  Consideration should be given to the potential consequences for the student and others.  For example, is the skill required during university study (i.e. study skills, such as searching sources for information), and the extent to which an underdeveloped skill might adversely affect colleagues or patients (e.g. timekeeping).  Depending on the curriculum, this is also likely to dictate a different priority given to different skills at different stages of education and training.  Different learning opportunities are likely to develop some skills more than others, thus a combination of repeated and alternate formats may be required to ensure that the necessary skills are achieved at each stage.

## Tip 3 - Tailor training to the needs of each student

Students will have different starting strengths and weaknesses, and different personal perspectives of the value and their own capacity to develop different skills.   While the target is to achieve at least the minimum requirements, knowledge of different individual starting points can enable identification of different personal development needs.  There can be significant differences in background and training experiences prior to receiving any formal medical education ( Whittle and Murdoch-Eaton, 2004), and graduate students will also have different needs to undergraduate students.  It is possible to map each student’s ability and aptitude in each required transferable skill (e.g. establishing predisposition through self-evaluation).  Self-evaluation is needed to calibrate self-confidence in transferable skill abilities to a realistic level ( Whittle and Murdoch-Eaton, 2002) and recognise that not everyone will have the same aptitude for all skills.  Thus, it is important to manage expectations, build confidence, provide personalised learning opportunities and review.  Framing expectations is particularly important (e.g. to avoid the notion that ‘I didn’t come here to learn this, this isn’t medicine’) especially if the skill is seen as generic and ‘bolted-on’, with summative implications on a par with clinical acumen.  It is important to consider ways that we can help students understand the need for transferable skills in comparison to specific clinically relevant knowledge and skills (e.g. why they have to do reflective writing), and to generate motivation through relevance to practice.  A framework for helping students to ‘learn to learn’ at university has been proposed ( Wingate, 2007), in which students are coached in a structured way from the earliest stage (before induction if possible) in key learning skills. The authors suggest that this should be provided for all students in a proactive, rather than remedial, manner. They argue that we should be quite explicit with the students in terms of the skills they need to acquire, and that we must teach them in a subject specific context ( Wingate, 2007).  Thus it is necessary for teaching staff to know the stage the students are at and the skills they need to be developing.  It is important to encourage students to reflect and think about what it is they want to develop; all the time highlighting the clinical context so that they can see the purpose.

## Tip 4 – Combine with a degree of student choice

Confirmation of transferable skill outcomes can be combined with student selected components.  Transferable skills outcomes need to be adopted as core underlying themes and summatively assessed to ensure that mandatory requirements are met.  This is easier to achieve where skills are assessed independently of, and clearly emphasised relative to, expert content knowledge.  Often the aims of courses emphasise transferable skills, whilst the students are assessed mainly on factual recall, which reduces the motivation to learn the skills ( Momsen et al., 2010).  The extent of mastery in mandatory skills, or development of additional optional skills, provides further opportunity for student selection.  Providing the opportunity to develop different skills to different degrees of mastery by choice may help to: explore suitability for a future career aspiration (e.g. as a clinical academic), to improve a weakness, or build on a strength ( Whittle and Murdoch-Eaton, 2001).  Thus, ensure that the emphasis is on the required transferable skills rather than expert content knowledge.  Then ensure that students are completely aware of what is being assessed, at what level, what the standard requirements are at each stage, and what additional targets they may choose.

## Tip 5 - Provide opportunities to develop at appropriate stages

Consider the order of learning.  Different abilities will be required at different points in education and training, and some skills will need to be revisited spirally (e.g. to build entrustability; Peters et al., 2017).  Thus, it is important to align provision of teaching and assessment to points of transition; surveying pre-existing skills at entry ( McLean et al., 2011) and providing appropriate orientation and induction.  Furthermore, the frequency (both number and spacing) of opportunities to learn and practice, and whether repetition or spiralling is appropriate, may vary with each transferable skill. Reflection is something that medical students are required to do at many points during their training. In the early years of study, this might be reflection on how they have worked with peers in a group, or how they have improved their study skills. This level of reflection may then help them to deal with more difficult situations later on, for example when dealing with patients or conflict with other clinicians.  Spiralling reflective practice in this way gives students the skills to be more resilient when they meet challenges in the future.  Be cognisant of student backgrounds and their starting abilities.  For example, written assignments may spiral from a requirement to: use patient information, write patient guidance information, and then write staff guidance and protocols; or to use referral letters, write a referral letter, and then respond to a complaint about one.

## Tip 6 - Authentically align skills to relevant workplace activities and methods

While by definition every transferable skill ( Table 1) has a degree of transferability on a spectrum from medical to non-medical applications, it is crucial that the clinical relevance is prioritised to ensure appropriate student motivation ( Berkhout et al., 2018).  Depending on the outcome required, it may be useful to separate the skill from its context (e.g. remedial instruction in study skills), or to combine the skill with its application within simulation or practice (e.g. entrustable activities).  Teaching skills in multiple relevant contexts helps students to realise the significance of the application ( Murdoch-Eaton and Whittle, 2012). For example, students will generally be expected to work in many different teams during their medical education. They will take different roles in these teams depending on whom they are working with. They may lead teams of their peers, but be expected to work to goals set by a team of clinicians or researchers. Similarly, with communication skills, placements offer students many chances to communicate with different audiences. This allows them to hone their skills in varied contexts with people having different, and sometimes conflicting, personalities; thus giving them first-hand experience of how important effective communication is in a clinical setting ( NHS, 2018).

## Tip 7 - Integrate training into the curriculum to an appropriate extent

Learning opportunities can be embedded (implicitly taught, without direct reference), integrated (explicitly taught), or bolted-on (taught as a separate extra; Chadha, 2005).  Some skills are better covered overtly as a transparent objective, either alongside other types of outcome or standalone, whereas others may be better covered more covertly combined within another activity. Skills like communication and networking may be best addressed by placing students in situations where they need to, for example, explain concepts to patients or work with clinicians. These types of skills are very context dependent and it is difficult to provide the student with a lesson on the rules for dealing with a particular situation, therefore integration might be the best approach. On the other hand, analysing evidence from clinical trials may be best taught in a more explicit way. We can explain to the students what the different statistical terms mean and can teach them about sample size and selection bias.  Therefore, it is important to consider the extent each skill should be embedded, integrated, or covered separately.

## Tip 8 - Provide appropriate instruction and student-centred opportunities

Student-centred learning of transferable skills can help optimise engagement ( Fox and Dryden, 1991). In addition, encouraging students to reflect on learning can help them to identify the transferable skills that they have gained ( Jha et al., 2002). It is important that students are aware of their individual skill sets, as being able to articulate these is key when applying for clinical roles. This may be facilitated by including various opportunities to involve expert staff, peers, patients or other members of the public; particularly if developing an entrustable framework (e.g. trust when supervised directly, indirectly, unsupervised, to supervise, or to assess others; Peters et al., 2017).  In any case, there needs to be appropriate mechanisms to flag additional opportunities for developing transferable skills and to provide support where needed.

## Tip 9 - Develop student understanding of introversion and extroversion

Students need to be made aware of the need to nurture and refine interpersonal abilities through introspection.  It is their responsibility to reflect and determine the degree of their own requirements for developing interaction with, and reliance on, others, and it is our responsibility to teach in ways that are inclusive.  In medicine, this is particularly important, since a successful clinician must work well with patients and with other professionals. Being a doctor also requires a level of empathy and social responsibility not needed in many other professions. That is not to say all doctors must be extroverts, more that medical training must allow enough opportunity for students to work out how best they communicate and work with others. In fact, introverted students tend to get higher grades early on in medical school, despite scoring less highly on interpersonal assessments and feeling more stressed later on ( Davidson et al., 2015). A variety of teaching styles must be used so that both introverts and extroverts can thrive at medical school ( Davidson et al., 2015).  For example, when students are at a placement we should make sure that introverts get as much opportunity as extroverts to learn from the interactions. We should bear in mind that providers of external placements might be less aware of this, and require training and support to teach in an inclusive way.

## Tip 10 - Structure using constructive or deconstructive approaches as appropriate

Consider the method of learning; is it best to start with a whole and proceed to dissect it, or start with component parts and proceeding to build, or to take a hybrid of these two approaches as a means to understanding and development depends on the skill?  For example in typical undergraduate science degrees, research skills are taught in a constructive way. Students begin by learning basic science, then reading review articles, then addressing the primary research literature, before conducting their own novel research in the final year. In medicine, we often expect students near the start of a medical programme to evaluate primary research and come to novel conclusions, before they have carried out any real research of their own. Whilst some skills, perhaps communication or reflection, might be best taught by immersion in context, research skills might be best addressed in a more stepwise fashion, helping the students understand good study design and how research works, before asking them to carry out their own novel research.  Overall, reinforcement is required: Students who mostly perform poorly can still sometimes excel when faced with something that they have a degree of familiarity with.  Better students, when faced with an unfamiliar case, are able to transfer their knowledge and reasoning skills across to a new situation, as there is enough transferable skill to at least make a start on a problem.  Therefore the development of expertise may, at least in part, be attributed to unstable constructs becoming progressively stable ( Shauber et al., 2018; Cookson, 2018).  Again the best way to approach skills training will differ according to the skill and the setting.

## Tip 11 - Ensure implementation is guided by cost-analyses, sustainability and scalability

It is important to confirm that the strategy for teaching transferable skills is fit for purpose.  This includes reviewing and monitoring for changes in: requirements and expectations, perceptions of acceptability, and effectiveness ( Walsh et al., 2013). This is particularly pertinent in the UK at present, with the large increase in student numbers planned for the next two years ( DoH, 2017). Many UK medical schools will currently be assessing their curricula and considering if they will still be suitable for much larger cohorts. Given that several of the transferable skills we identify here have interpersonal elements, it is key that as numbers increase, we do not lose the interaction between students and their peers, clinicians, academics and patients that is vital in the development of these skills.  Whilst all the evidence should be evaluated whenever changes in delivery are being considered, priority should be given to achieving the Learning Outcomes.

## Tip 12 - Link to evidence throughout

It is now clear that most published educational methods for improving transferable skills work; the priority must be to identify which methods produce the most success for which outcomes ( Hattie, 2015). Schneider and Preckel (2017) have extensively evaluated the effect size of different teaching variables on achievement.  For example, educational approaches have been ranked (1-105) according to the available evidence of their effect size by Schneider and Preckel (2017).  It is clear that instruction in extracurricular study is highly correlated with gaining academic skills (ranked 29), and to a lesser extent with developing self-management skills (60).  Instruction in simulated or authentic settings helps develop problem-solving (35), and student-led motivation improves goal orientation and gains in new knowledge (69).  Overall, methods employing social interaction have the greatest effect sizes and methods employing communication technology have the smallest effect sizes ( Schneider and Preckel, 2017).  Reviewing curricula in light of evidence such as this provides the opportunity to facilitate targeted changes to optimise delivery for maximum learning.

## Conclusions

Transferable skills differ in terms of how tangible they are and how they should best be delivered in the curriculum. When learning outcomes are set, the available empirical evidence should be utilised and thought should be given to how the skills will actually be taught. Educators should also pay close attention to how the skills are introduced, and when. Not all skills can be fully addressed in the first year of training for example. Appropriate spiralling should be included, with opportunities to have several attempts at learning skills in varying contexts and, importantly, time to reflect in-between.  In order to successfully deliver transferable skills in our curricula, we must also be very aware that our students are not entirely a homogenous group. We need to understand their educational and social backgrounds, consider their expectations and beliefs and their individual differences in terms of aptitudes for different skills.

## Take Home Messages

With the advent of modern technology, the recall of content knowledge no longer has the primacy it once did. By giving more thought to how we teach transferable skills, it is hoped that our students will be more capable life-long learners and better prepared for life as a clinician.

## Notes On Contributors

Dr Helen R Watson is a Lecturer at Peninsula Medical School, University of Plymouth.

Dr Steven A Burr is an Associate Professor and Deputy Director of Assessment for Medicine and Dentistry at Peninsula Medical School, University of Plymouth.
