# Supplementary material for: Twelve tips for teaching twelve transferable skills
Source: MedEdPublish (2016). 2018 Aug 21;7:177. [Version 1] doi: 10.15694/mep.2018.0000177.1 (PMC10701797; doi:10.15694/mep.2018.0000177.1)
Supplement: Peer review history

# Reviewer report: Reviewer response for version 1

**DOI:** 10.21956/mep.19644.r29279

**Published:** 2018-08-28

**Authors:** Hui Meng Er

**Affiliations:** 1 International Medical University

This review has been migrated. The reviewer awarded 4 stars out of 5 This paper addresses the pertinent issues in teaching transferable skills to health professional students. It is written in a clear and systematic manner, and the tips are aligned with contemporary learning philosophies. The significance of transferable skills to doctors in an era of knowledge explosion is well explained in the Introduction. However, there is little information on how the twelve tips were identified and the context of how these came about. This information would be useful for those who are considering implementing similar learning outcomes in their curricula. As mentioned in Tip 1, one of the challenges with transferable skills is ensuring constructive alignment between developing the skills and their assessment. Some insights on how a balance between knowledge and transferable skills can be achieved in curriculum and assessment would be appreciated. Student-led activities, reflection and personalised learning have been suggested, these are very much aligned with the self-regulated learning principles. It would be useful to also discuss the practical aspects such as the roles of faculty and training required.

Reviewer Expertise:

NA

No decision status is available

# Reviewer report: Reviewer response for version 1

**DOI:** 10.21956/mep.19644.r29280

**Published:** 2018-08-21

**Authors:** Poh-Sun Goh

**Affiliations:** 1 National University of Singapore

This review has been migrated. The reviewer awarded 4 stars out of 5 1) The Title (of this article) caught my attention. This topic is clearly relevant to many of us (in our teaching practice, or as learners.2) The article is generally clearly written. And clearly grounded in experience of day to day practice (by the authors). The writing voice (of the authors) from the coal face and front lines of day to day teaching practice, and program design/assessment rings out.3) The following might add to the article:a) Clearly articulating value to students and trainees, by anchoring skill training in pressing/immediate, and important professional and clinical tasks, illustrated by case studies from professional practice (adding to Tip 1).b) I have personally found instructional models and paradigms useful, for example the 4C-ID model http://edutechwiki.unige.ch/en/4C-ID (and this could be touched upon without adding too much to article length).c) The See (Show relevance, Why before How idea), Do (Practice with feedback, scaffolding, support), Reflect (To course correct, iteratively improve, self-direct), Repeat process is one way of thinking about professional training. Capture attention, Show why knowledge, skill, attitudes/mindsets are relevant and of value; Then offer opportunities (curricular space and time as the authors illustrate) to practice.4) This article will add to thinking, and conversations we have (as academics and practitioners) on this important topic, and makes for a thoughtful read.

Reviewer Expertise:

NA

No decision status is available
